# ELDER ABUSE AMID THE OPIOID EPIDEMIC: APS CASES IN RURAL AMERICA

**DOI:** 10.1093/geroni/igz038.2803

**Published:** 2019-11-08

**Authors:** Pamela B Teaster, Karen A Roberto, Jyoti Savla

**Affiliations:** 1 Virginia Tech, Blacksburg, Virginia, United States; 2 Center for Gerontology, Virginia Tech, BLACKSBURG, Virginia, United States

## Abstract

Older adults are hidden victims of the opioid crises, suffering abuse at the hands of those who seek resources to support their addiction. Using APS data from 2015-2017 provided by the Kentucky Department for Community-Based Services, we used a logistic regression model with robust standard errors to examine whether substantiated cases of elder abuse were associated with opioid misuse by perpetrators. Overall, 9% of the 462 substantiated cases over the three-year period involved perpetrators were substance users. The percentage of these cases rose from 5% in 2015 to 13% in 2016 before dropping to 7% of elder abuse cases in 2017. Opioid use was most prevalent among perpetrators of financial abuse of older adults with cognitive and/or physical care needs. The current study offers a first look at empirical linkages between opioid misuse and elder abuse and revealed consistencies across cases that call for further investigation.

